# Assessing Treatment Effects with Pharmacometric Models: A New Method that Addresses Problems with Standard Assessments

**DOI:** 10.1208/s12248-021-00596-8

**Published:** 2021-05-03

**Authors:** Estelle Chasseloup, Adrien Tessier, Mats O. Karlsson

**Affiliations:** 1 Department of Pharmacy, Uppsala University, Uppsala, Sweden; 2 Division of Quantitative Pharmacology, Institut de Recherches Internationales Servier, Suresnes, France

**Keywords:** type I error, bias, drug effect, nonlinear mixed effect models, mixture models

## Abstract

Longitudinal pharmacometric models offer many advantages in the analysis of clinical trial data, but potentially inflated type I error and biased drug effect estimates, as a consequence of model misspecifications and multiple testing, are main drawbacks. In this work, we used real data to compare these aspects for a standard approach (STD) and a new one using mixture models, called individual model averaging (IMA). Placebo arm data sets were obtained from three clinical studies assessing ADAS-Cog scores, Likert pain scores, and seizure frequency. By randomly (1:1) assigning patients in the above data sets to “treatment” or “placebo,” we created data sets where any significant drug effect was known to be a false positive. Repeating the process of random assignment and analysis for significant drug effect many times (*N* = 1000) for each of the 40 to 66 placebo-drug model combinations, statistics of the type I error and drug effect bias were obtained. Across all models and the three data types, the type I error was (5th, 25th, 50th, 75th, 95th percentiles) 4.1, 11.4, 40.6, 100.0, 100.0 for STD, and 1.6, 3.5, 4.3, 5.0, 6.0 for IMA. IMA showed no bias in the drug effect estimates, whereas in STD bias was frequently present. In conclusion, STD is associated with inflated type I error and risk of biased drug effect estimates. IMA demonstrated controlled type I error and no bias.

**Supplementary Information:**

The online version contains supplementary material available at 10.1208/s12248-021-00596-8.

## INTRODUCTION

The use of nonlinear mixed effect models has become a standard in drug development as evidenced by best practice documents generated by companies (1, 2) and guidances issued by regulatory agencies (3, 4). While the use of models initially focused on the characterization of pharmacokinetics in the patient population, it is nowadays used to answer a multitude of questions. Among these, relationship between treatment and response is of particular interest. In an assessment of the value brought to the organization from various modeling activities, model-based characterization of the treatment effect is featured in a majority of the examples (5). With adequate models, model-based analyses of longitudinal data can typically provide analyses where the treatment effects can be more precisely estimated and identified with much higher power compared to analyses of end-of-treatment data (6, 7). Despite these potential advantages, model-based analyses represent a minority of the analyses that address the primary questions about treatment effects in drug development (8). Concerns regarding model selection bias and model misspecification are primary impediments for the more widespread use of model-based analyses.

A data-driven model-building process can lead to model selection bias that exaggerates the treatment effect. To avoid such problems, model averaging based on a preselected set of drug effect models has been suggested (9–11). These are promising techniques to address the issue of selection bias; however, the problem of model misspecification with model-based analyses may be aggravated from the selection among a small number of predefined models. The main concerns regarding model misspecification include the risk of a biased drug effect estimate and the incorrect conclusion that a drug is efficacious, when it is not. The latter is typically quantified as the type I error rate of a test where the null hypothesis is no drug effect and the alternative is a drug effect associated with treatment. Several investigations have focused on the gains from model-based analyses when drug effects are present, but fewer have addressed the risk of incorrect conclusions regarding the treatment effect when there is no drug effect. In this work, we try to assess such risks. While there are many designs in which treatment effect characterization is the primary objective, we focus here on a simple parallel group two-arm design where the aim is to compare treatment with placebo. The main evaluations from such a study are the drug effect estimate and whether this estimate is statistically significantly different from zero. Additional evaluations to assess commercial viability may also be included (12) but will not be the focus here.

In addition to evaluating the standard (STD) modeling strategy for assessing drug effects, based on the treatment arm having an estimated component, the drug effect, absent in the placebo arm, we will also introduce and assess a new approach, “individual model averaging” (IMA). In this approach, two submodels (placebo alone and placebo plus drug effect) are simultaneously fitted to each individual’s data. The probability for individuals to be explained by one or the other submodel is then estimated through a mixture model, using the allocation arm (placebo or treatment) as predictor. The standard full model is a special case of this model, namely, when the estimated probability to be described by the placebo alone model is one in the placebo arm and zero in the treatment arm.

To study the impact of model misspecification, we utilized real data examples where all patients received the same treatment, placebo or standard of care. This was preferred over simulating data, as there is typically a limited understanding on the origin of model misspecification and it is therefore difficult to realistically simulating it. With real data where all participants receive placebo, it is possible to mimic trials of “treatment” versus “placebo” by random arm allocation for each patient. Doing so, each subject is assigned an arm in a trial for which appropriate analyses should not indicate differences between “treatment” and “placebo” beyond what can happen by chance.

## METHODS

NONMEM version 7.4.3 and PsN version 4.9.1 were used to analyze the clinical data, and the post-processing of the results was performed with R version 3.6.0. The estimation methods used were FOCE for the continuous data and LAPLACE for the categorical data.

### Data

The comparisons between the STD and the IMA methods were performed using three placebo data sets: ADAS-Cog scores treated as continuous data, categorical Likert pain score, and seizures count data. For each data set, three different designs were created using different number of subjects. Table I presents a summary of the data used in this study.

**Table I:** Summary of the Placebo Data Sets Used for the Different Designs in This Study

Data sets	Number of subjects^a^	Total observation count	Follow-up duration^b^
	800	3519	24 months (1–36)
ADAS-cog	400	1732	24 months (1–36)
	200	894	24 months (1–36)
	230	3226	119 days (0–119)
Likert pain score	120	1671	119 days (0–119)
	60	788	119 days (0–119)
	500	6397	77 days (21–224)
Seizure	250	3135	77 days (49–168)
	50	646	77 days (63–154)

^*a*^Randomly sampled from the initial data set

^*b*^Median (min–max)

#### ADAS-Cog

The ADAS-cog (Alzheimer’s Disease Assessment Scale–Cognitive) scale scores the severity of the disease between 0 and 70. Due to the high number of categories, the data were considered as continuous. The data were collected from the ADNI data base (www.adni-info.org); a detailed description is available elsewhere (13). The recruited individuals were aged between 55 and 90 years old: 229 cognitively normal elderly, 188 presenting early Alzheimer disease, and 405 with mild cognitive impairment. The follow-up duration was respectively 3, 2, and 3 years, with ADAS-cog evaluation at 0, 6, 12, 24, and 36 months. For convenience, the data set was reduced to 800 individuals.

#### Likert Pain Score

The Likert pain score is an 11-point pain scale ranging from 0 to 10. The data were recorded from 231 patients with painful distal diabetic neuropathy randomized in the placebo arm of three phase III studies. Pain scores and allowed intake of acetaminophen, a rescue medicine, were recorded daily. A description of the data initially gathered is available elsewhere (14, 15). We reduced the data to weekly records, keeping an even number of individuals (230).

#### Seizure

The seizure count data included daily seizures count from patients (551) with medically refractory partial seizures, obtained during the screening phase (12 weeks), of a larger study where each patient was screened maintaining his standard antiepileptic treatment (same brand, formulation, and dosage) (16). We reduced the data to weekly records, keeping an even number of individuals (500).

### Definition of the STD and IMA Methods

In both methods, the likelihood ratio test (LRT) discriminates between the null hypothesis (*H*_0_), i.e. a base model without the treatment information, and the alternative hypothesis (*H*_1_), i.e. a full model accounting for the treatment information. The two approaches are summarized in Table II, and code examples are given in the electronic supplemental material ESM 1.

**Table II:** Null and Alternative Hypothesis Description for the Standard and the Individual Model Averaging Approaches

Approach	Null hypothesis (H_0_)	Alternative hypothesis (H_1_)
**STD**	*BASE* + *PLB* (Eq. 1)	*BASE* + *PLB* + *DRUG* × *ARM* (Eq. 2)
**IMA**	Submodel 1: *BASE* + *PLB* + *DRUG* (Eq. 3) Submodel 2: *BASE* + *PLB* (Eq. 1)	
	*P*(1) = 0.5 *P*(2) = 1 − *P*(1)	*P*(1)= *ARM* ∙ (1 − *θ*_*mix*_) + (1 − *ARM*) ∙ *θ*_*mix*_ *P*(2) = 1 − *P*(1)

*STD*, standard approach; *IMA*, individual model averaging approach; *BASE*, baseline model; *PLB*, placebo model; *DRUG*, drug model; *P(1)*, probability of allocation to submodel 1; *P(2)*, probability of allocation to submodel 2; *θ*_*mix*_, estimated mixture probability

#### Standard Method

This approach is the current method used to test if a treatment has a significant impact on a clinical outcome, compared to another intervention such as placebo or standard of care. In this approach, the base model describes the observations without specific parameter for the studied treatment intervention (Eq. 1), and the full model adds a drug effect model to quantify the effect of the studied treatment intervention based on the arm assignment (Eq. 2):

1 $$ y=b\left({\theta}_B,{\eta}_B,\gamma \right)\diamond p\left(t,{\theta}_{PLB},{\eta}_{PLB},\gamma \right) $$

2 $$ y=b\left({\theta}_B,{\eta}_B,\gamma \right)\diamond p\left(t,{\theta}_{PLB},{\eta}_{PLB},\gamma \right)\diamond d\left(t,{\theta}_{DRUG},{\eta}_{DRUG},\gamma, ARM\right) $$

where *y* is the individual predictions for continuous data and the probability of observing a given discrete event for categorical data. *b* is the function describing the observations at baseline, *p* is the function describing their evolution without treatment effect (i.e. placebo model), *d* is the the function describing the impact of the treatment (i.e. drug model), and ⋄ any arithmetic operation (addition or multiplication). *t* is the time; *θ*_*X*_ and *η*_*X*_, respectively, are the fixed and random effects of the functions; γ is the covariate vector; and *ARM* is the binary assignment with the individual treatment information: 0 when not treated, 1 when treated.

#### IMA Method

In the individual model averaging approach, a mixture model is used to describe the data for each patient via two submodels: submodel 1, with drug effect (Eq. 3), and submodel 2, without drug effect (Eq. 1). In the base model, the probability of each subject is given by the study randomization (*P*(1) = *P*(2) = 0.5, where *P*(1) and *P*(2) are the probabilities for submodel 1 and 2, respectively). In the full model, this proportion is estimated conditioned on the arm assignment (Eq. 4):

3 $$ y=b\left({\theta}_B,{\eta}_B,\gamma \right)\diamond p\left(t,{\theta}_{PLB},{\eta}_{PLB},\gamma \right)\diamond d\left(t,{\theta}_{DRUG},{\eta}_{DRUG},\gamma \right) $$

4 $$ \Big\{{\displaystyle \begin{array}{c}P(1)= ARM\cdot {\theta}_{mix}+\left(1- ARM\right)\cdot \left(1-{\theta}_{mix}\right)\\ {}P(2)=1-P(1)\end{array}}\operatorname{} $$

where ARM ∈ {0, 1} according to the randomization and θ_mix_ ∈[0, 1] is the estimated mixture proportion. For a mixture model with *n* individuals and *m* subpopulations (*m* = 2 for IMA), the objective function value (OFV) is the sum of the individual likelihood (*IL*_*i*_), computed weighting the individual likelihood of each mixture submodel (*IL*_*i*, *k*_) with the mixture proportion (*P*_*pop*, *k*_) (17):

5 $$ OFV=\sum \limits_{i=1}^n OF{V}_i=\sum \limits_{i=1}^n-2\ln \left(I{L}_i\right) $$

6 $$ I{L}_i=\sum \limits_{k=1}^mI{L}_{i,k}\cdotp {P}_{pop,k}=\sum \limits_{k=1}^m\exp \left(- OF{V}_{i,k}/2\right)\cdotp {P}_{pop,k} $$

where *i* is the *i*th individual with *i* ∈ {1, 2, …, n} and *k* is the *k*th submodel with *k* ∈ {1, 2}.

### Type I Error Computation

The type I errors of the LRT between the full and base model for the STD and the IMA approach were investigated using the real placebo data sets. To compute the type I error rate, the individuals were randomized (1:1) repeatedly (*N* = 1000) between a placebo reference arm and a treated arm, creating *N* permutations of the random bivariate assignment vector (0, non-treated arm, and 1, treated arm), mimicking *N* parallel group studies controlled with placebo. The number of degrees of freedom used for the LRT was the number of parameters estimated in the drug model for the STD approach, whereas it was 1 in IMA, corresponding to the estimation of the mixture proportion. The type I error rate was assumed to be adequate if it was within the 2.5th and 97.5th percentiles of a binomial distribution with a probability of success of 5% on N=1000 trial replicates: [3.73-6.54].

This procedure was repeated with different combinations of placebo and drug effect models (40 combinations for the Likert pain score and the seizure count data, 66 for the ADAS-cog data), to explore the robustness of the methods towards model misspecifications. The equations corresponding to the placebo and the drug model used for each data set are detailed in the electronic supplemental material ESM 1.

### Drug Effect Estimates

The computation of the bias and the precision in the drug estimates was adapted to the drug effect parameterization and the approach. The typical drug effect computation was using the typical drug estimates (*θ*_*drug*_) only for STD, whereas for IMA it was a function of both the typical drug estimates and the mixture proportion (*θ*_*mix*_) as described in Table III. A *θ*_*mix*_ estimate of 0.5 (0 or 1) implied no relationship (a strong relationship) between the arm assignment and the submodel probability which only differ by the presence of a drug effect.

**Table III:** Computation of Drug Effect Estimates

Drug model	Data	Drug effect parameterization	Typical drug effect
Offset	Likert pain score	*BASE* + *PLB* + (*θ*_*drug*_ + *η*_*drug*_)	(*θ*_*mix*_ ∗ 2 − 1) ∗ *θ*_*drug*_
	Seizure count	$BASE\ast PLB\ast {e}^{\left({\theta}_{drug}+{\eta}_{drug}\right)}$	(*θ*_*mix*_ ∗ 2 − 1) ∗ *θ*_*drug*_
	ADAS-cog	*BASE* + *PLB* − (*θ*_*drug*_ + *η*_*drug*_)	(*θ*_*mix*_ ∗ 2 − 1) ∗ *θ*_*drug*_
Linear	Likert pain score	*BASE* + *PLB* + (*θ*_*drug*_ + *η*_*drug*_) ∗ *t*	(*θ*_*mix*_ ∗ 2 − 1) ∗ *θ*_*drug*_ ∗ *t*_*last*_
	Seizure count	$BASE\ast PLB\ast {e}^{\left({\theta}_{drug}+{\eta}_{drug}\right)\ast t}$	(*θ*_*mix*_ ∗ 2 − 1) ∗ *θ*_*drug*_ ∗ *t*_*last*_
	ADAS-cog	*BASE* + *PLB* − (*θ*_*drug*_ + *η*_*drug*_) ∗ *PLB*	(*θ*_*mix*_ ∗ 2 − 1) ∗ *θ*_*drug*_ ∗ *PLB*

*BASE*, baseline model; *PLB*, placebo model; *θ*_*drug*_, drug effect parameter; *η*_*drug*_, drug effect inter-individual variability normally distributed (0 for models without inter-individual variability); *θ*_*mix*_, mixture probability (1 for the standard approach); *t*_*last*_, time at the end of the study

## RESULTS

**Fig. 1:**
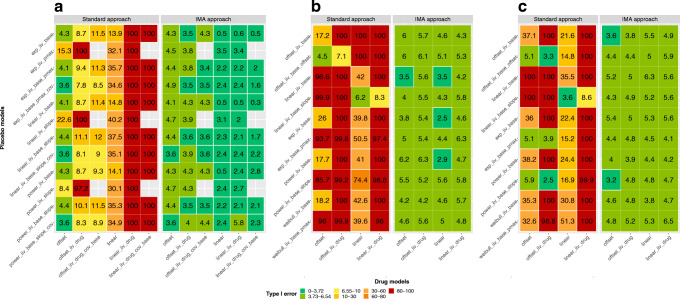
Comparison of type I error between the standard (STD) and the individual model averaging (IMA) approaches for different combination of placebo and drug models, with continuous ADAS-cog data (**a**), categorical Likert pain score data (**b**), and seizure count data (**c**)

Figure 1 showed that across all three data sets, the type I error rate of the STD approach (minimum, 25th, 50th, 75th percentiles, and maximum, 2.5, 11.4, 40.6, 100.0, 100.0) was high and not controlled, contrary to the type I error rate of the IMA approach (0.3, 3.5, 4.3, 5.0, 6.5). Additional information regarding the fit of these models (base model OFV, mean full model OFV, and number of successful minimizations) are provided in the electronic supplemental material ESM 3.

For the STD approach, the false positive rate was the highest with the Likert pain score data (4.5, 40.7, 97.0, 100.0, 100.0), the lowest with the ADAS-cog data (3.6, 8.8, 26.4, 100.0, 100.0), and the seizure count data had intermediate performances (2.5, 16.4, 37.6, 100.0, 100.0). For the ADAS-cog data, the type I error rate was minimum with the offset drug model and maximum with the linear drug models including inter-individual variability (IIV). The placebo model without IIV on baseline had worse performances when combined with the offset drug models with IIV. For the Likert pain score data, the models (placebo or drug) adding a second IIV parameter resulted in worst type I error performances, the offset placebo model with IIV combined with offset drug models, and linear placebo model with IIV combined with linear drug models being the only exceptions.

For the seizure count data, the drug models including IIV tended to have worse type I error. The offset drug model with IIV had a better controlled type I error when used with a placebo model including two IIV parameters. The use of the linear placebo model with two IIV parameters performed poorly in combination with offset drug models but had better performance when combined with the linear drug model with IIV and a controlled type I error in combination with a linear drug model.

For the IMA approach, the type I error was well controlled, 2.5, 4.3, 5.1, 5.6, and 6.3, for the Likert pain score data; 3.2, 4.4, 4.8, 5.2, and 6.5 for the seizure count data; and for most combinations lower than the 5% confidence interval for the ADAS-cog data (0.3, 2.2, 3.5, 4.3, 5.8). None of the placebo drug model combination tested for the three data sets had a type I error above the 95th confidence interval of the 0.05 proportion (3.73–6.54%), but 43/66, 4/40, and 2/40 were below it for the ADAS-cog data, the Likert pain score, and the seizure count data, respectively. For the ADAS-cog data, the combinations including a linear drug model had lower type I error rates. For the Likert pain score data, all the combinations with a very low type I error included a drug model without IIV.

**Fig. 2:**
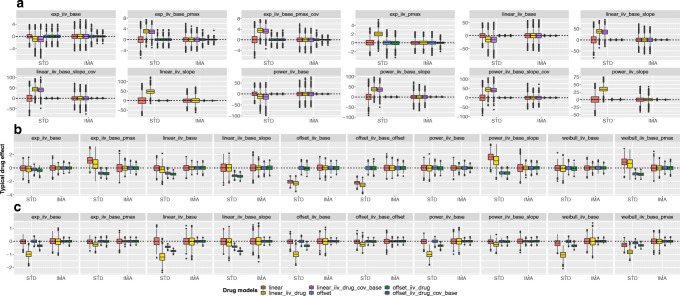
Comparison of bias and precision in drug effect estimates between the standard (STD) and the individual model averaging (IMA) approaches for different combination of placebo and drug models, with continuous dADAS-cog data (**a**), categorical Likert pain score data (**b**), and seizure count data (**c**). The plot is facetted by placebo models

Figure 2 illustrates the bias and the precision of the drug effect estimates. With the IMA approach, no bias was observed in the typical value of the drug effect estimate, and the offset models provided a better precision. For the STD approach, the precision had a similar magnitude as the IMA approach, but the typical value was often biased. The bias was observed only with the linear drug models for the ADAS-cog data but independently of the model used for the Likert pain score and the seizure count data.

## DISCUSSION

The results showed the overall superiority of IMA over STD: STD presented inflated type I error and generally biased typical drug effects, whereas IMA had controlled type I error and unbiased typical drug effects. Similar results were observed for smaller sample sizes of the same data sets (see electronic supplemental material ESM 4).

The hypotheses tested using the STD and IMA approaches differ, and the results can be understood from these differences. For STD, the hypothesis test answers the question whether an additional model component can significantly improve description of the treatment arm data. It does not answer the question whether there is a systematic difference between the data in the treatment and placebo arms. In STD, it is assumed, but not tested, that the same additional model component would not improve the description of the data in the placebo arm. The inflated type I error of the STD approach, and the bias in the drug effect estimates, can thus be explained by misspecification of the placebo models combined with an ability of the drug effect model to improve the description of data even in the absence of a true drug effect. Thus, in the absence of a true drug effect, a significant test may result from any situation where the drug effect component can compensate for a misspecified placebo model. Naturally, this probability increases with the inadequacy of the placebo model and the ability of the drug effect model to compensate for this inadequacy. It can be seen in the results that drug effect models that have the same functional form as the placebo models lead to less inflation of the type I error. The ability to compensate is less when the functional form of the drug model is the same as for placebo model. In addition, it can be seen that the more flexible drug effect models that include IIV more often lead to higher type I errors.

For IMA, the hypothesis test answers the question whether, without changing the model structure, the treatment allocation information can improve the description of the data. Thus, it is expected that if there is no systematic difference between data in the two arms, the test would not show any inflation in the type I error rate regardless of model misspecifications. This is also the finding of our study, where in no case, type I errors are inflated or drug effect estimates are biased, not even when the drug model clearly can improve the description of data above that of the placebo model. Such properties of a modeling approach are always desirable but especially so for situations where the model(s) to be used needs to be predefined as is typically the case for primary analyses in order to avoid subjective model-building.

The full IMA model estimates one more parameter, θ_mix_, than the full STD model. There are two considerations that extend from the STD approach: setting of initial estimates and calculation of drug effect from the model parameter estimates. A natural set initial estimates for the full IMA model are the final estimates from the base IMA model together with an initial estimate of θ_mix_ at 0.5. Thus, no new parameters need to be introduced, and the starting point assures that the fit is at least as good as the base IMA model. An alternative, complementary, starting point for the full IMA model would be a model close to the full STD model by setting θ_mix_ to a value close to one and other parameters as in the full STD model. In this case, a fit at least as good as the full STD model is assured. The calculation of the estimated drug effect from the full IMA effect always requires consideration of θ_mix_ in addition to the standard drug effect parameters. In the simplest case, this can be done as outlined in Table III, but in the presence of more complex drug effect models and/or the calculations of drug effect metrics that also integrates variability parameters, this may require additional calculations.

For both STD and IMA approaches, the LRT was used to test for the presence of a treatment effect. The LRT assumes that, in the absence of a drug effect, the difference in OFV between the reduced and the full models follows a chi-squared distribution of *n* degrees of freedom, *n* being the difference in number of parameters between the two models. For the STD approach, the addition of a parameter with the drug model does not always correspond to an additional full degree of freedom. Examples of such situations are parameters set at a boundary in the base model (e.g., variance parameters), nonlinear functions (e.g., sigmoid Emax model), redundancies between parameters (e.g., disease-modifying drug effect when disease progression is negligible), or boundaries in the parameter space. A consequence when having fewer actual than nominal degrees of freedom is a lower type I error rate and lower power. With the IMA approach, the only difference between the two models is the estimation of the mixture proportion, θ_mix_, in the full model, conditioned on the arm allocation. Hence, the comparison between the full and base IMA models constitutes a full degree of freedom. Only in the case when the two submodels are the same, or essentially the same, would the comparison constitute less than a full degree of freedom. An example when the two submodels are almost the same is for ADAS-Cog when the linear, disease-modifying, effect is applied and the disease progression rate is close to zero. It can be noted that the type I error in those cases is systematically below the nominal 5% level (Fig. 1a, IMA approach). For the avoidance of misunderstanding, note that the situation studied here is quite different from the addition of a covariate effect on a parameter. In this case, only post-baseline predictions will be influenced, and also in other respects, these additional functions often contain time dependencies. The results presented here should therefore not be interpreted as informative on type 1 error and estimation bias for standard parameter-covariate relations.

The ability of drug effect models to improve the data description, because of model misspecification in the placebo model, is the reason for the inflated type I error and biased drug effect estimate of STD in the studied examples. The drug effect model typically provides additional flexibility in the description of the time-course of the response; therefore, defects in the placebo model in the description of the response time-course would be the most concerning feature. In relation to this investigation, if the placebo models used were too simple and/or the drug effect models unusually complex or tailored to capitalize on the placebo model misspecification, the results may not be representative. However, we believe the placebo time-course models tested are representative of common practice. Further, standard goodness-of-fit assessments for the best of the placebo models tried in this study for each data set indicate acceptable data description (see visual predictive checks and goodness-of-fit plots in the electronic supplemental material ESM 2). It should be noted that also for those “best” placebo models, type I error was inflated. Further, the drug models of this investigation were also tested on the published models and data sets according to the standard approach, and the result show inflation of type I error rate (see electronic supplemental material ESM 5). For the Likert pain score data, the published model run time was not manageable; hence, the dOFV (difference in OFV) for one randomization is shown instead. These results show also that the inclusion of significant covariates (ADAS-cog data) or over-dispersion parameter (seizure count data) did not decrease the type I error rate. The published data set of the seizure count has daily records contrary to the weekly data used in this investigation; hence, richer data did not help to decrease the type I error. Finally, as shown by Tessier et al, in a context without model misspecification (simulated data), no type I error inflation was observed for STD. These conclusions strongly suggest for model misspecification as the culprit for the observed type I error inflation with the STD approach.

The current study focused on type I error of combination of placebo and drug models without the context of multiple testing that often happens during the model-building step. As multiple testing is also a major cause of type I error inflation, it is interesting to note that in the case of STD, it is a negligible problem because of the very high type I error rate of the placebo and drug model combinations individually. However, since IMA showed a controlled type I error at this level, the type I error inflation due to multiple testing is expected to be significant. It is hence advisable to follow the health authorities’ guidelines on the topic which recommends a priori model specification to avoid multiple testing and its caveats. Nonetheless, it can be noted that since, contrary to STD, IMA showed robustness towards model misspecification, it should not be impacted by model pre-specification. Further, IMA allows assessment of different placebo and drug effect models without introducing information on the treatment allocation. The full IMA could then be compared to the refined base IMA model. Whether such model refinement also inflates type I error is another line of investigation that could be performed.

While dose–response or exposure–response analyses are more common in pharmacometrics, this study focuses on treatment–response analysis. It is the most basic settings to test for drug effect, hence a good starting point to assess a new method which should be able to recognize the absence of drug effect in such scenario. Dose– or exposure–response models can enhance the power of the pharmacometric analysis compared to treatment–response models. Nonetheless, regarding the type I error, when working on data without drug effect, drug– or exposure–response analyses are not expected to lower the type I error compared to treatment response analyses.

**Fig. 3:**
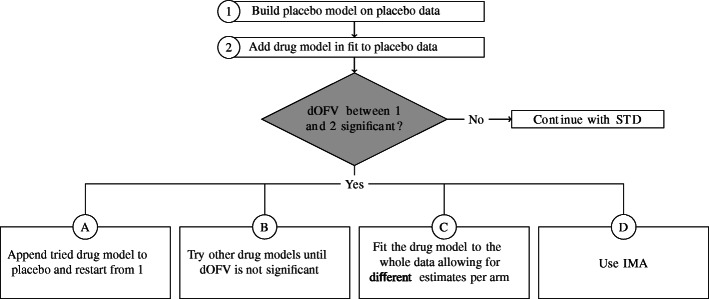
Alternative workflow for the standard approach. STD, standard approach; IMA, individual model averaging; dOFV, difference in objective function value

While the IMA approach appears promising and deserving further investigation, the results also point to modifications, or variants, of the STD approach that seem warranted. If we consider one variant of the STD approach as (1) build a placebo model on the placebo data, then (2) fix the placebo parameters and build the drug model with the drug data, and (3) estimate all the parameters with the whole data set. This process is subject to selection bias in the drug effect estimate because of the multiple models tried, especially in step 2, and to model misspecification, which always is present. The latter problem has different consequences depending on the part of the model impacted: misspecifications in the placebo model are likely to inflate the type I error, while misspecifications in the drug model are likely to decrease the power to identify a drug effect, and misspecifications in either are likely to bias drug effect estimates. The contrast between the performances of STD and IMA suggests some good practices when it comes to modeling drug effect using the STD approach. Figure 3 outlines alternative workflows, such as fitting drug models on placebo data to check for placebo model misspecification as described in A and B or allowing the estimation of the “drug” effect in all arms with different values as described in C and D.

This investigation focused on the use of STD and IMA for assessing treatment effects in the situation of analysis of real data when there is no drug effect present. This focus was selected as concern for falsely identifying a positive treatment effect is an important hindrance for use of pharmacometric models in primary analyses. A complementary question of interest is how the power of identifying an existing drug effect compares between the STD and IMA approaches. There are also many other aspects of implementation of the IMA framework in drug development that would benefit from study. These situations include the use in studies with other designs, such as different allocation ratio, multiple arm studies like dose–response studies, or studies with crossover components. Further, the incorporation of IMA in other types of analyses, such as exposure–response or “classical” model averaging (9–11), may require additional considerations. All these related investigations have been pursued and will be reported separately.

## CONCLUSIONS

The STD approach is associated with inflated type I error and risk of biased drug effect estimates. This is a consequence of placebo model misspecification. As model misspecification always occurs, standard model-building procedures ought to incorporate a step in which the drug model’s ability to improve the fit to placebo data is explored.

The IMA approach demonstrated controlled type I error and no bias. These are desirable properties in any model-based analysis that could prove to be particularly important when a model-based analysis is to serve as a primary analysis and form the basis for decisions in drug development or drug usage.

## Supplementary Information

ESM 1 (DOCX 32 kb)

ESM 2 (DOCX 2743 kb)

ESM 3 (DOCX 1229 kb)

ESM 4 (DOCX 922 kb)

ESM 5 (DOCX 73 kb)

## References

[1] EFPIA MID3 Workgroup, Marshall SF, Burghaus R, Cosson V, Cheung SYA, Chenel M, et al. Good Practices in Model-Informed Drug Discovery and Development: Practice, Application, and Documentation. CPT Pharmacometrics Syst Pharmacol. 2016;5:93–122. 10.1002/psp4.12049 PMC4809625 27069774

[2] Marshall S, Madabushi R, Manolis E, Krudys K, Staab A, Dykstra K, Visser SAG (2019). Model-informed drug discovery and development: Current industry good practice and regulatory expectations and future perspectives. CPT Pharmacometrics Syst Pharmacol.

[3] Center for Drug Evaluation and Research (CDER), Center for Biologics Evaluation and Research (CBER). FDA guidance for industry: Exposure-response relationships — Study design, data Analysis, and regulatory applications [Internet]. U.S. Department of Health and Human Services, Food and Drug Administration; 2003 [cited 2020 Mar 3]. Available from: https://www.fda.gov/media/71277/download.

[4] Office of Medical Products and Tobacco, Center for Drug Evaluation and Research, Office of Medical Products and Tobacco, Center for Biologics Evaluation and Research. FDA guidance for industry: Population pharmacokinetics [Internet]. Food and Drug Administration; 2019 [cited 2020 Mar 3]. Available from: http://www.fda.gov/regulatory-information/search-fda-guidance-documents/population-pharmacokinetics.

[5] Milligan PA, Brown MJ, Marchant B, Martin SW, van der Graaf PH, Benson N, Nucci G, Nichols DJ, Boyd RA, Mandema JW, Krishnaswami S, Zwillich S, Gruben D, Anziano RJ, Stock TC, Lalonde RL (2013). Model-based drug development: A rational approach to efficiently accelerate drug development. Clin Pharmacol Ther.

[6] Ueckert S, Plan EL, Ito K, Karlsson MO, Corrigan B, Hooker AC (2014). Improved utilization of ADAS-cog assessment data through item response theory based pharmacometric modeling. Pharm Res.

[7] Karlsson KE, Vong C, Bergstrand M, Jonsson EN, Karlsson MO (2013). Comparisons of analysis methods for proof-of-concept trials. CPT Pharmacometrics Syst Pharmacol.

[8] Lee JY, Garnett CE, Gobburu JVS, Bhattaram VA, Brar S, Earp JC, Jadhav PR, Krudys K, Lesko LJ, Li F, Liu J, Madabushi R, Marathe A, Mehrotra N, Tornoe C, Wang Y, Zhu H (2011). Impact of pharmacometric analyses on new drug approval and labelling decisions. Clin Pharmacokinet.

[9] Aoki Y, Röshammar D, Hamrén B, Hooker AC (2017). Model selection and averaging of nonlinear mixed-effect models for robust phase III dose selection. J Pharmacokinet Pharmacodyn.

[10] Buatois S, Ueckert S, Frey N, Retout S, Mentré F (2018). Comparison of model averaging and model selection in dose finding trials analyzed by nonlinear mixed effect models. AAPS J.

[11] Dosne AG, Bergstrand M, Karlsson MO, Renard D, Heimann G (2017). Model averaging for robust assessment of QT prolongation by concentration-response analysis. Stat Med.

[12] Kirby S, Chuang-Stein C (2017). A comparison of five approaches to decision-making for a first clinical trial of efficacy. Pharm Stat.

[13] Ito K, Corrigan B, Zhao Q, French J, Miller R, Soares H, Katz E, Nicholas T, Billing B, Anziano R, Fullerton T, Alzheimer's Disease Neuroimaging Initiative (2011). Disease progression model for cognitive deterioration from Alzheimer’s disease neuroimaging initiative database. Alzheimers Dement.

[14] Plan EL, Elshoff J-P, Stockis A, Sargentini-Maier ML, Karlsson MO (2012). Likert pain score modeling: a Markov integer model and an autoregressive continuous model. Clin Pharmacol Ther.

[15] Schindler E, Karlsson MO (2017). A minimal continuous-time Markov pharmacometric model. AAPS J.

[16] Trocóniz IF, Plan EL, Miller R, Karlsson MO (2009). Modelling overdispersion and Markovian features in count data. J Pharmacokinet Pharmacodyn.

[17] Carlsson KC, Savić RM, Hooker AC, Karlsson MO (2009). Modeling subpopulations with the $MIXTURE subroutine in NONMEM: Finding the individual probability of belonging to a subpopulation for the use in model analysis and improved decision making. AAPS J.

