# Supplementary material for: Assessing Treatment Effects with Pharmacometric Models: A New Method that Addresses Problems with Standard Assessments
Source: AAPS J. 2021 May 3;23(3):63. doi: 10.1208/s12248-021-00596-8 (PMC8093168; doi:10.1208/s12248-021-00596-8)
Supplement: Supplementary file 1 — (DOCX 32 kb) [file 12248_2021_596_MOESM1_ESM.docx]

**Supplementary material 1: definitions for placebo and drug models, and NONMEM code example for the standard and the individual model averaging approach.**

[Table 1: Definition of the models used for the ADAS-cog data 2](#_Toc48834042)

[Table 2: Definition of the models used for the Likert pain score data 3](#_Toc48834043)

[Table 3: Definition of the models used for the seizures count data 4](#_Toc48834044)

[Table 4: NONMEM code example for the standard approach 5](#_Toc48834045)

[Table 5: NONMEM code example for the individual model averaging approach 6](#_Toc48834046)

Table 1: Definition of the models used for the ADAS-cog data

| Code | Description | Formula |
| --- | --- | --- |
| PLACEBO MODELS | | |
| exp_iiv_base | Exponential with IIV on baseline | ${TV}_{BASE}+\eta_{BASE}+\theta_{PMAX}*\left( 1-{exp}^{-\frac{\log\left( 2 \right)}{\theta_{HL}}t} \right)$ |
| exp_iiv_pmax | Exponential with IIV on pmax | ${TV}_{BASE}+{(\theta}_{PMAX}+\eta_{PMAX})*\left( 1-{exp}^{-\frac{\log\left( 2 \right)}{\theta_{HL}}t} \right)$ |
| exp_iiv_base_pmax | Exponential with IIV on baseline and pmax | ${TV}_{BASE}+\eta_{BASE}+{(\theta}_{PMAX}+\eta_{PMAX})*\left( 1-{exp}^{-\frac{\log\left( 2 \right)}{\theta_{HL}}t} \right)$ |
| exp_iiv_base_pmax_cov | Exponential with IIV on baseline and pmax, and covariance between baseline and pmax | ${TV}_{BASE}+\eta_{BASE}+{(\theta}_{PMAX}+\eta_{PMAX})*\left( 1-{exp}^{-\frac{\log\left( 2 \right)}{\theta_{HL}}t} \right)$ |
| linear_iiv_base | Linear with IIV on baseline | ${TV}_{BASE}+\eta_{BASE}+\theta_{SLOPE}*t$ |
| linear_iiv_slope | Linear with IIV on slope | ${TV}_{BASE}+{(\theta}_{SLOPE}+\eta_{SLOPE})*t$ |
| linear_iiv_base_slope | Linear with IIV on baseline and slope | ${TV}_{BASE}+\eta_{BASE}+{(\theta}_{SLOPE}+\eta_{SLOPE})*t$ |
| linear_iiv_base_slope_cov | Linear with IIV on baseline and slope, and covariance between baseline and slope | ${TV}_{BASE}+\eta_{BASE}+{(\theta}_{SLOPE}+\eta_{SLOPE})*t$ |
| power_iiv_base | Power with IIV on baseline | ${TV}_{BASE}+\eta_{BASE}+\theta_{SLOPE}*t^{\theta_{POWER}}$ |
| power_iiv_slope | Power with IIV on slope | ${TV}_{BASE}+{(\theta}_{SLOPE}+\eta_{SLOPE})*t^{\theta_{POWER}}$ |
| power_iiv_base_slope | Power with IIV on baseline and slope | ${TV}_{BASE}+\eta_{BASE}+{(\theta}_{SLOPE}+\eta_{SLOPE})*t^{\theta_{POWER}}$ |
| power_iiv_base_slope_cov | Power with IIV on baseline and slope, and covariance between baseline and slope | ${TV}_{BASE}+\eta_{BASE}+{(\theta}_{SLOPE}+\eta_{SLOPE})*t^{\theta_{POWER}}$ |
| DRUG MODELS | | |
| offset | Offset | $\theta_{DRUG}$ |
| offset_iiv_drug | Offset with IIV on the drug effect | $\theta_{DRUG}+\eta_{DRUG}$ |
| offset_iiv_drug_cov_base | Offset with IIV on the drug effect, and covariance between baseline and drug effect | $\theta_{BASE}+\eta_{BASE}$ |
| linear | Linear | $PLACEBO*(1- \theta_{DRUG})$ |
| linear_iiv_drug | Linear with IIV on the drug effect | $PLACEBO*(1- \theta_{DRUG}+\eta_{DRUG})$ |
| linear_iiv_drug_cov_base | Linear with IIV on the drug effect, and covariance between baseline and drug effect | $PLACEBO*(1- \theta_{DRUG}+\eta_{DRUG})$ |

$TV_{\mathrm{BASE}}$: typical value for the baseline, $\mathrm{PLACEBO}$: placebo model without the baseline parameters; $\theta$: fixed effects parameters; $\eta$: random effects parameters following $\eta\sim\mathcal{N}(0,\sigma^{2})$; pmax: maximum placebo effect; $HL$: half-life; and $t$: the time.

Table 2: Definition of the models used for the Likert pain score data

| Code | Description | Formula |
| --- | --- | --- |
| PLACEBO MODELS | | |
| offset_iiv_base | Offset with IIV on baseline | $BASE+\theta_{OFFSET}$ |
| offset_iiv_base_offset | Offset with IIV on baseline and offset | $BASE+\theta_{OFFSET}+\eta_{OFFSET}$ |
| linear_iiv_base | Linear with IIV on baseline | $BASE+\theta_{SLOPE}*t$ |
| linear_iiv_base_slope | Linear with IIV on baseline and slope | $BASE+{(\theta}_{SLOPE}+\eta_{SLOPE})*t$ |
| exp_iiv_base | Exponential with IIV on baseline | $BASE+\theta_{PMAX}* \left( 1-{exp}^{-\frac{\log\left( 2 \right)}{\theta_{HL}}t} \right)$ |
| exp_iiv_base_pmax | Exponential with IIV on baseline and pmax | $BASE+\left( \theta_{PMAX}+\eta_{PMAX} \right)* \left( 1-{exp}^{-\frac{\log\left( 2 \right)}{\theta_{HL}}t} \right)$ |
| power_iiv_base | Power with IIV on baseline | $BASE+\theta_{SLOPE}*t^{\theta_{POWER}}$ |
| power_iiv_base_slope | Power with IIV on baseline and slope | $BASE+{(\theta}_{SLOPE}+\eta_{SLOPE})*t^{\theta_{POWER}}$ |
| weibull_iiv_base | Weibull with IIV on baseline | $BASE+\theta_{PMAX}* \left( 1-{exp}^{-{(\frac{\log\left( 2 \right)}{\theta_{HL}}t)}^{\theta_{POWER}}} \right)$ |
| weibull_iiv_base_pmax | Weibull with IIV on baseline and pmax | $BASE+\left( \theta_{PMAX}+\eta_{PMAX} \right)* \left( 1-{exp}^{-{(\frac{\log\left( 2 \right)}{\theta_{HL}}t)}^{\theta_{POWER}}} \right)$ |
| DRUG MODELS | | |
| offset | Offset | $\theta_{DRUG}$ |
| offset_iiv_drug | Offset with IIV on the drug effect | $\theta_{DRUG}+\eta_{DRUG}$ |
| linear | Linear | $\theta_{DRUG}*t$ |
| linear_iiv_drug | Linear with IIV on the drug effect | $\left( \theta_{DRUG}+\eta_{DRUG} \right)*t$ |

$BASE=\theta_{BASE}+\eta_{BASE}$, the baseline parameterization; $\theta$: fixed effects parameters; $\eta$: random effects parameters following $\eta\sim\mathcal{N}(0,\sigma^{2})$; pmax: maximum placebo effect; $HL$: half-life; and $t$: the time.

Table 3: Definition of the models used for the seizures count data

| Code | Description | Formula |
| --- | --- | --- |
| PLACEBO MODELS | | |
| offset_iiv_base | Offset with IIV on baseline | $BASE*\theta_{OFFSET}$ |
| offset_iiv_base_offset | Offset with IIV on baseline and offset | $BASE*\theta_{OFFSET}*\eta_{OFFSET}$ |
| linear_iiv_base | Linear with IIV on baseline | $BASE*\theta_{SLOPE}*t$ |
| linear_iiv_base_slope | Linear with IIV on baseline and slope | $BASE*{(\theta}_{SLOPE}+\eta_{SLOPE})*t$ |
| exp_iiv_base | Exponential with IIV on baseline | $BASE*\theta_{PMAX}* \left( 1-{exp}^{-\frac{\log\left( 2 \right)}{\theta_{HL}}t} \right)$ |
| exp_iiv_base_pmax | Exponential with IIV on baseline and pmax | $BASE*\left( \theta_{PMAX}+\eta_{PMAX} \right)* \left( 1-{exp}^{-\frac{\log\left( 2 \right)}{\theta_{HL}}t} \right)$ |
| power_iiv_base | Power with IIV on baseline | $BASE*\theta_{SLOPE}*t^{\theta_{POWER}}$ |
| power_iiv_base_slope | Power with IIV on baseline and slope | $BASE*{(\theta}_{SLOPE}+\eta_{SLOPE})*t^{\theta_{POWER}}$ |
| weibull_iiv_base | Weibull with IIV on baseline | $BASE*\theta_{PMAX}* \left( 1-{exp}^{-{(\frac{\log\left( 2 \right)}{\theta_{HL}}t)}^{\theta_{POWER}}} \right)$ |
| weibull_iiv_base_pmax | Weibull with IIV on baseline and pmax | $BASE*\left( \theta_{PMAX}+\eta_{PMAX} \right)* \left( 1-{exp}^{-{(\frac{\log\left( 2 \right)}{\theta_{HL}}t)}^{\theta_{POWER}}} \right)$ |
| DRUG MODELS | | |
| offset | Offset | $\theta_{DRUG}$ |
| offset_iiv_drug | Offset with IIV on the drug effect | $\theta_{DRUG}+\eta_{DRUG}$ |
| linear | Linear | $\theta_{DRUG}*t$ |
| linear_iiv_drug | Linear with IIV on the drug effect | $\left( \theta_{DRUG}+\eta_{DRUG} \right)*t$ |

$BASE=\theta_{BASE}+\eta_{BASE}$, the baseline parameterization; $\theta$: fixed effects parameters; $\eta$: random effects parameters following $\eta\sim\mathcal{N}(0,\sigma^{2})$; pmax: maximum placebo effect; $HL$: half-life; and $t$: the time.

Table 4: NONMEM code example for the standard approach

| STD approach | |
| --- | --- |
| Base model | Full model |
| $PROBLEM STD base model  $INPUT ID TIME DV BMMS ARM  $DATA cleaned.csv IGNORE=@  $ABBREVIATED COMRES=3  $PRED  ;----BASELINE MODEL-----  INTERCEPT = THETA(1)  BSLP = THETA(2)  TVBSL = INTERCEPT + BSLP * BMMS  BASELINE = TVBSL + ETA(1)  ;----PLACEBO MODEL-----  PMAX = THETA(5)  HL = THETA(6)  PLACEBO = PMAX*(1-EXP(-LOG(2)/HL*TIME))  ;----DRUG MODEL-----  **DRUG = 0**  ADASCOG = BASELINE + DRUG + PLACEBO  IF(TIME.EQ.0)  ADASCOG = BASELINE + PLACEBO  $THETA (0,52.6) ; PRM INTER 1  $THETA -1.59 ; PRM BSLP 2  $THETA (0,3.25) ; PRM EPS 3  $THETA 1.0 ; PRM PMAX 4  $THETA (0,9.97,100) ; PRM HL 5  ;  $OMEGA 0.09 ; PRM OMBASE 1  $SIGMA 1 FIX  $ESTIMATION MAXEVAL=9999 METHOD=1 NOABORT NOTHETABOUNDTEST  NOOMEGABOUNDTEST | $PROBLEM STD full model  $INPUT ID TIME DV BMMS ARM  $DATA cleaned.csv IGNORE=@  $ABBREVIATED COMRES=3  $PRED  ;----BASELINE MODEL-----  INTERCEPT = THETA(1)  BSLP = THETA(2)  TVBSL = INTERCEPT + BSLP * BMMS  BASELINE = TVBSL + ETA(1)  ;----PLACEBO MODEL-----  PMAX = THETA(5)  HL = THETA(6)  PLACEBO = PMAX*(1-EXP(-LOG(2)/HL*TIME))  ;----DRUG MODEL-----  **DRUG = THETA(6)*ARM**  ADASCOG = BASELINE + DRUG + PLACEBO  IF(TIME.EQ.0)  ADASCOG = BASELINE + PLACEBO  $THETA (0,52.6) ; PRM INTER 1  $THETA -1.59 ; PRM BSLP 2  $THETA (0,3.25) ; PRM EPS 3  $THETA 1.0 ; PRM PMAX 4  $THETA (0,9.97,100) ; PRM HL 5  **$THETA (0,0.098,1000000) ; PRM DRUG 6**  $OMEGA 0.09 ; PRM OMBASE 1  $SIGMA 1 FIX  $ESTIMATION MAXEVAL=9999 METHOD=1 NOABORT NOTHETABOUNDTEST  NOOMEGABOUNDTEST |

The differences between the base and the full models are highlighted using a bold font. The differences between the standard and the IMA approach are highlighted in gray.

Table 5: NONMEM code example for the individual model averaging approach

| Base model | Full model |
| --- | --- |
| $PROBLEM IMA base model  $INPUT ID TIME DV BMMS ARM  $DATA cleaned.csv IGNORE=@  $ABBREVIATED COMRES=3  $PRED  ;----BASELINE MODEL-----  INTERCEPT = THETA(1)  BSLP = THETA(2)  TVBSL = INTERCEPT + BSLP * BMMS  BASELINE = TVBSL + ETA(1)  ;----PLACEBO MODEL-----  PMAX = THETA(5)  HL = THETA(6)  PLACEBO = PMAX*(1-EXP(-LOG(2)/HL*TIME))  ;----DRUG MODEL-----  DRUG = THETA(7)  IF(MIXNUM.EQ.1)  ADASCOG = BASELINE + PLACEBO + DRUG  IF(MIXNUM.EQ.2)  ADASCOG = BASELINE + PLACEBO  IF(TIME.EQ.0)  ADASCOG = BASELINE + PLACEBO  $CONTR DATA=(ARM)  $MIX  NSPOP = 2  ; ARM = 0: placebo  ; ARM = 1: treatment  PMIX1 = THETA(4)  **P(1) = PMIX1**  P(2) = 1 - P(1)  $THETA (0,62.106) ; PRM INTER 1  $THETA -1.88048 ; PRM BSLP 2  $THETA (0,3.13154) ; PRM EPS 3  **$THETA 0.5 FIX ; PRM MIX 3**  $THETA 1.87587 ; PRM PMAX 5  $THETA (0,5.69015,100) ; PRM HL 6  $THETA (0,620.012,1000000) ; PRM DRUG 7  $OMEGA 20.1959 ; PRM OMBASE 1  $SIGMA 1 FIX  $ESTIMATION MAXEVAL=9999 METHOD=1 NOABORT NOTHETABOUNDTEST  NOOMEGABOUNDTEST | $PROBLEM IMA full model  $INPUT ID TIME DV BMMS ARM  $DATA cleaned.csv IGNORE=@  $ABBREVIATED COMRES=3  $PRED  ;----BASELINE MODEL-----  INTERCEPT = THETA(1)  BSLP = THETA(2)  TVBSL = INTERCEPT + BSLP * BMMS  BASELINE = TVBSL + ETA(1)  ;----PLACEBO MODEL-----  PMAX = THETA(5)  HL = THETA(6)  PLACEBO = PMAX*(1-EXP(-LOG(2)/HL*TIME))  ;----DRUG MODEL-----  DRUG = THETA(7)  IF(MIXNUM.EQ.1)  ADASCOG = BASELINE + PLACEBO + DRUG  IF(MIXNUM.EQ.2)  ADASCOG = BASELINE + PLACEBO  IF(TIME.EQ.0)  ADASCOG = BASELINE + PLACEBO  $CONTR DATA=(ARM)  $MIX  NSPOP = 2  ; ARM = 0: placebo  ; ARM = 1: treatment  PMIX1 = THETA(4)  **P(1) = PMIX1*ARM + (1-PMIX1)*(1-ARM)**  P(2) = 1 - P(1)  $THETA (0,62.106) ; PRM INTER 1  $THETA -1.88048 ; PRM BSLP 2  $THETA (0,3.13154) ; PRM EPS 3  **$THETA (0,0.5,1) ; PRM MIX 3**  $THETA 1.87587 ; PRM PMAX 5  $THETA (0,5.69015,100) ; PRM HL 6  $THETA (0,620.012,1000000) ; PRM DRUG 7  $OMEGA 20.1959 ; PRM OMBASE 1  $SIGMA 1 FIX  $ESTIMATION MAXEVAL=9999 METHOD=1 NOABORT NOTHETABOUNDTEST  NOOMEGABOUNDTEST |

The differences between the base and the full models are highlighted using a bold font. The differences between the standard and the IMA approach are highlighted in gray.
