# Supplementary material for: Assessing Treatment Effects with Pharmacometric Models: A New Method that Addresses Problems with Standard Assessments
Source: AAPS J. 2021 May 3;23(3):63. doi: 10.1208/s12248-021-00596-8 (PMC8093168; doi:10.1208/s12248-021-00596-8)
Supplement: Supplementary file 2 — (DOCX 2743 kb) [file 12248_2021_596_MOESM2_ESM.docx]

**Supplemental material 2: Goodness of fit plots for the best placebo models**

No VPCs is shown for the best placebo model (according to the OFV) of the ADAS-cog data since a dropout model was missing, even for the published model, hence the VPCs were inappropriate to assess model adequacy.

[Figure 1: Conditional weighted residuals for the best placebo model (exp_iiv_base_pmax_cov) of the ADAS-cog data. 1](#_Toc64384100)

[Figure 2: Categorical visual predictive checks per score for the best placebo model (exp_iiv_base_pmax) of the Likert-pain score data, including 1000 simulations. Black line and dots represent the observed proportion of individuals having the score. The blue shaded area represents the corresponding 95% confidence interval of the simulations. 2](#_Toc64384101)

[Figure 3: Continuous visual predictive checks for the best placebo model (exp_iiv_base_pmax) of the Likert-pain score data, including 1000 simulations. Black lines represent the 50](#_Toc64384102)^[th](#_Toc64384102)^ [(solid line), 2.5](#_Toc64384102)^[th](#_Toc64384102)^ [and 97.5](#_Toc64384102)^[th](#_Toc64384102)^ [(dashed lines) observations percentiles. The shaded areas represent the corresponding 95% confidence interval of the simulations. Empty blue circles represent the observations. 2](#_Toc64384102)

[Figure 4: Pearson weighted residuals](#_Toc64384103)^[1](#_Toc64384103)^ [for the best placebo model (exp_iiv_base_pmax) of the Likert-pain score data. 3](#_Toc64384103)

[Figure 5: Categorical visual predictive checks per count for the best placebo model (weibull_iiv_base_pmax) of the seizures count data, including 1000 simulations. Black line and dots represent the observed proportion of individuals having the seizure count. The blue shaded area represents the 95% confidence interval of the simulations. 3](#_Toc64384104)

[Figure 6: Pearson weighted residuals](#_Toc64384105)^[1](#_Toc64384105)^ [for the best placebo model (weibull_iiv_base_pmax) of the seizures count data. 4](#_Toc64384105)

Figure 1: Conditional weighted residuals for the best placebo model (exp_iiv_base_pmax_cov) of the ADAS-cog data.


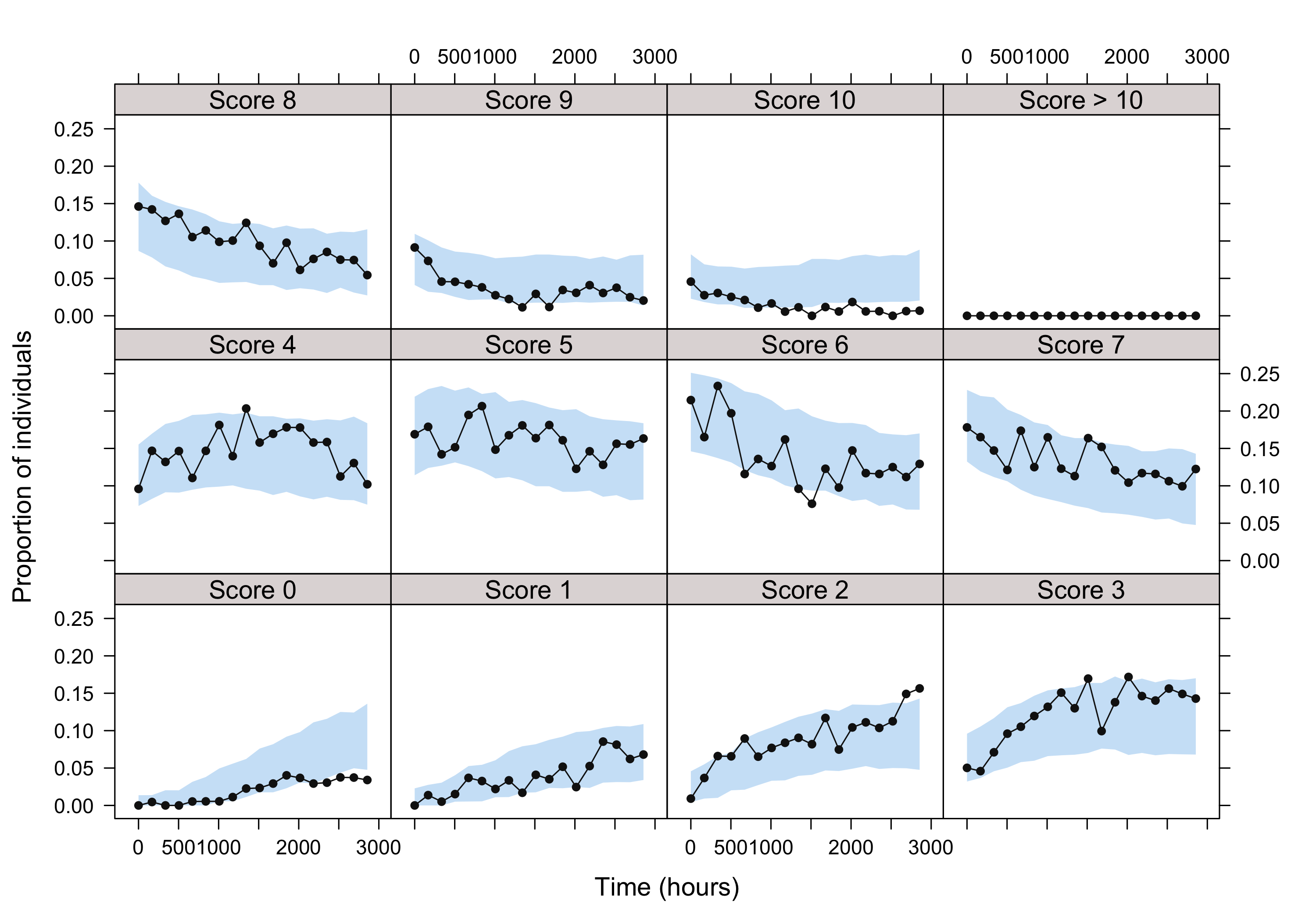


Figure 2: Categorical visual predictive checks per score for the best placebo model (exp_iiv_base_pmax) of the Likert-pain score data, including 1000 simulations. Black line and dots represent the observed proportion of individuals having the score. The blue shaded area represents the corresponding 95% confidence interval of the simulations.


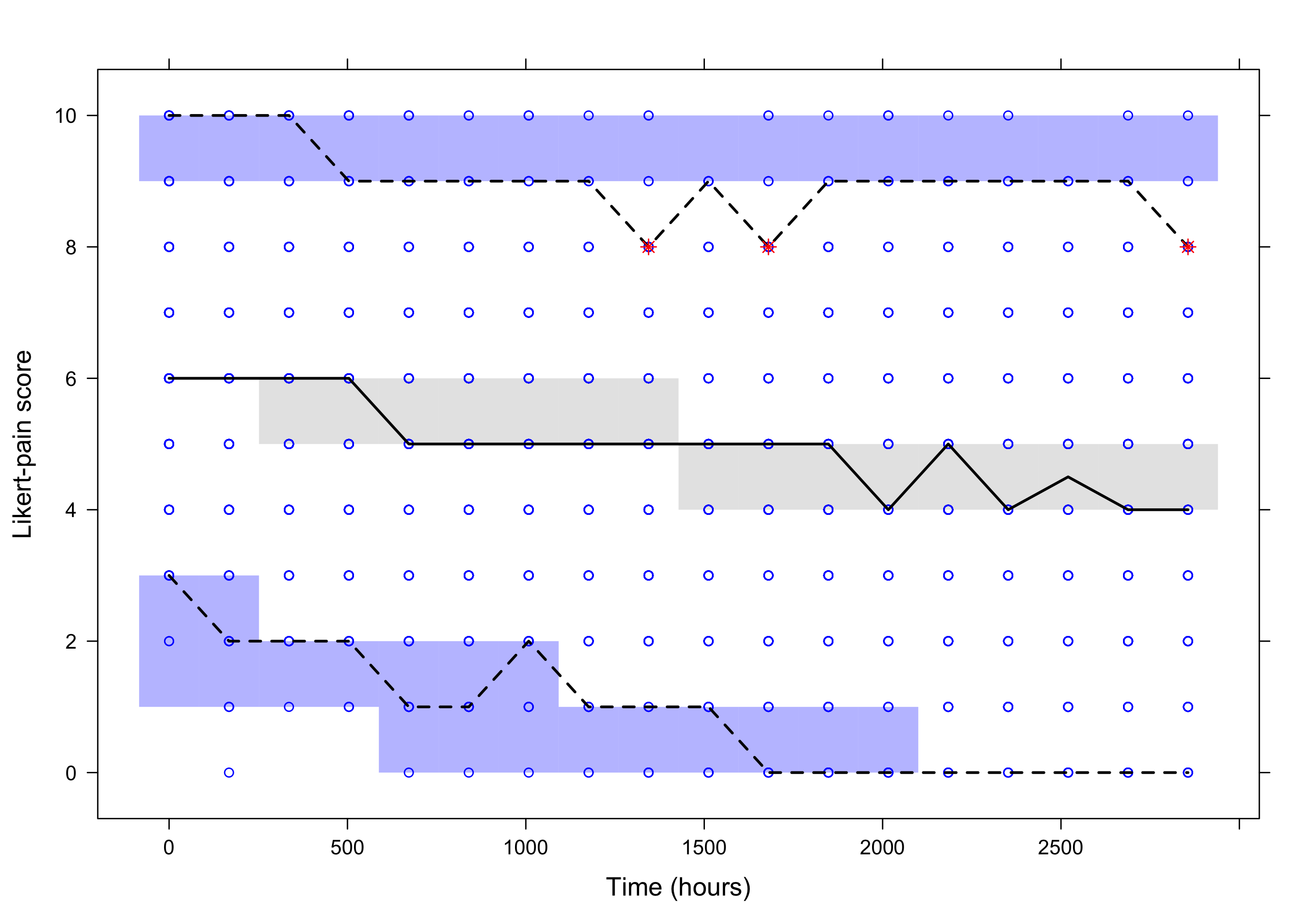


Figure 3: Continuous visual predictive checks for the best placebo model (exp_iiv_base_pmax) of the Likert-pain score data, including 1000 simulations. Black lines represent the 50^th^ (solid line), 2.5^th^ and 97.5^th^ (dashed lines) observations percentiles. The shaded areas represent the corresponding 95% confidence interval of the simulations. Empty blue circles represent the observations.

Figure 4: Pearson weighted residuals^1^ for the best placebo model (exp_iiv_base_pmax) of the Likert-pain score data.


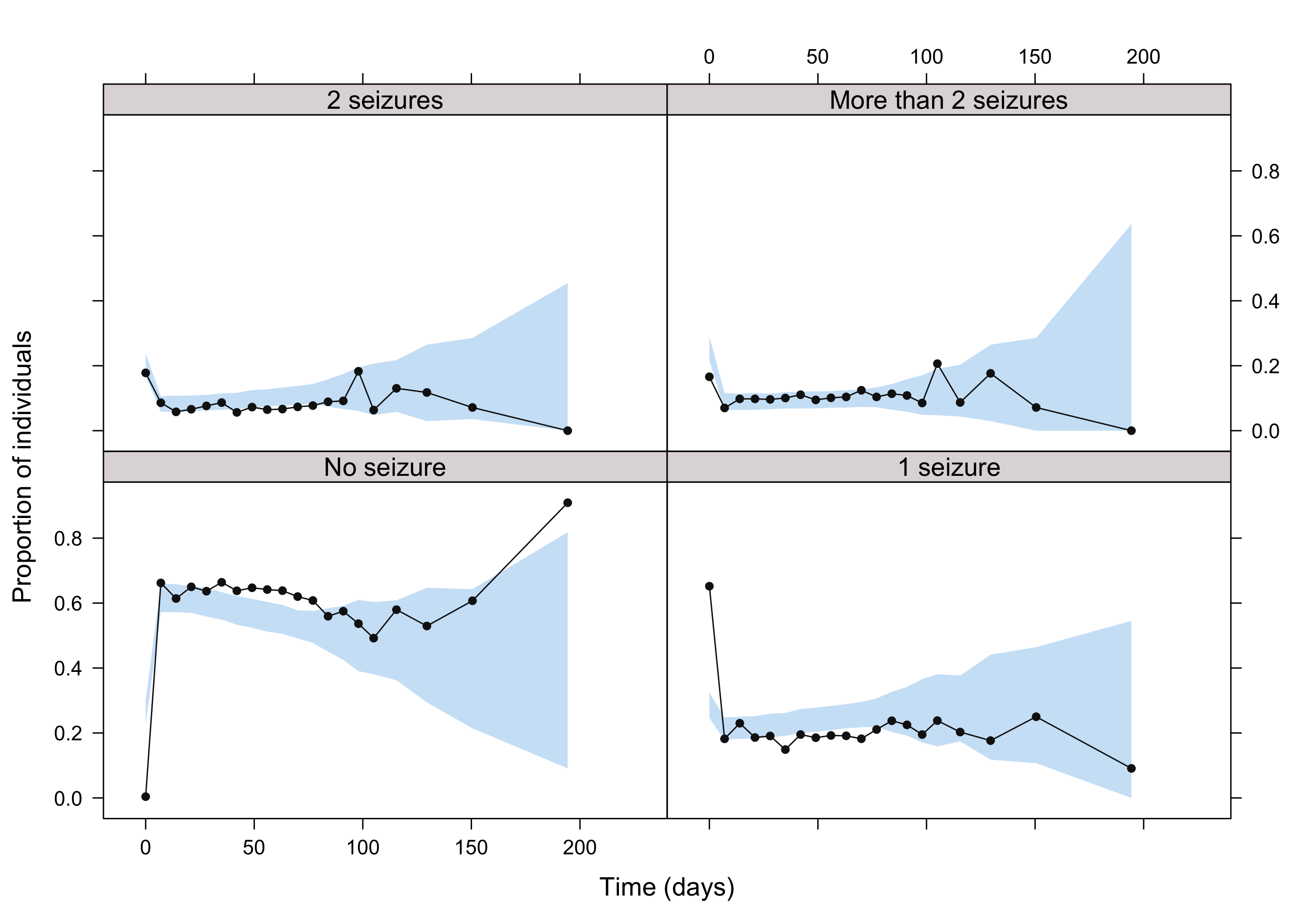


Figure 5: Categorical visual predictive checks per count for the best placebo model (weibull_iiv_base_pmax) of the seizures count data, including 1000 simulations. Black line and dots represent the observed proportion of individuals having the seizure count. The blue shaded area represents the 95% confidence interval of the simulations.

Figure 6: Pearson weighted residuals^1^ for the best placebo model (weibull_iiv_base_pmax) of the seizures count data.

^1^Wellhagen GJ, Kjellsson MC, Karlsson MO. A bounded integer model for rating and composite scale data. The AAPS journal. 2019 Jul;21(4):1-8
