# Supplementary material for: Assessing Treatment Effects with Pharmacometric Models: A New Method that Addresses Problems with Standard Assessments
Source: AAPS J. 2021 May 3;23(3):63. doi: 10.1208/s12248-021-00596-8 (PMC8093168; doi:10.1208/s12248-021-00596-8)
Supplement: Supplementary file 3 — (DOCX 1229 kb) [file 12248_2021_596_MOESM3_ESM.docx]

**Supplemental material 3: Additional details about models fitted.**

[Figure 1: Successful minimization, OFV of the base model, and dOFV between the base OFV and the average full OFV for the ADAS-cog data 2](#_Toc48842197)

[Figure 2: Successful minimization, OFV of the base model, and dOFV between the base OFV and the average full OFV for the Likert-pain score data 3](#_Toc48842198)

[Figure 3: Successful minimization, OFV of the base model, and dOFV between the base OFV and the average full OFV for the seizure count data 4](#_Toc48842199)

Figure 1: Successful minimization, OFV of the base model, and dOFV between the base OFV and the average full OFV for the ADAS-cog data

Figure 2: Successful minimization, OFV of the base model, and dOFV between the base OFV and the average full OFV for the Likert-pain score data


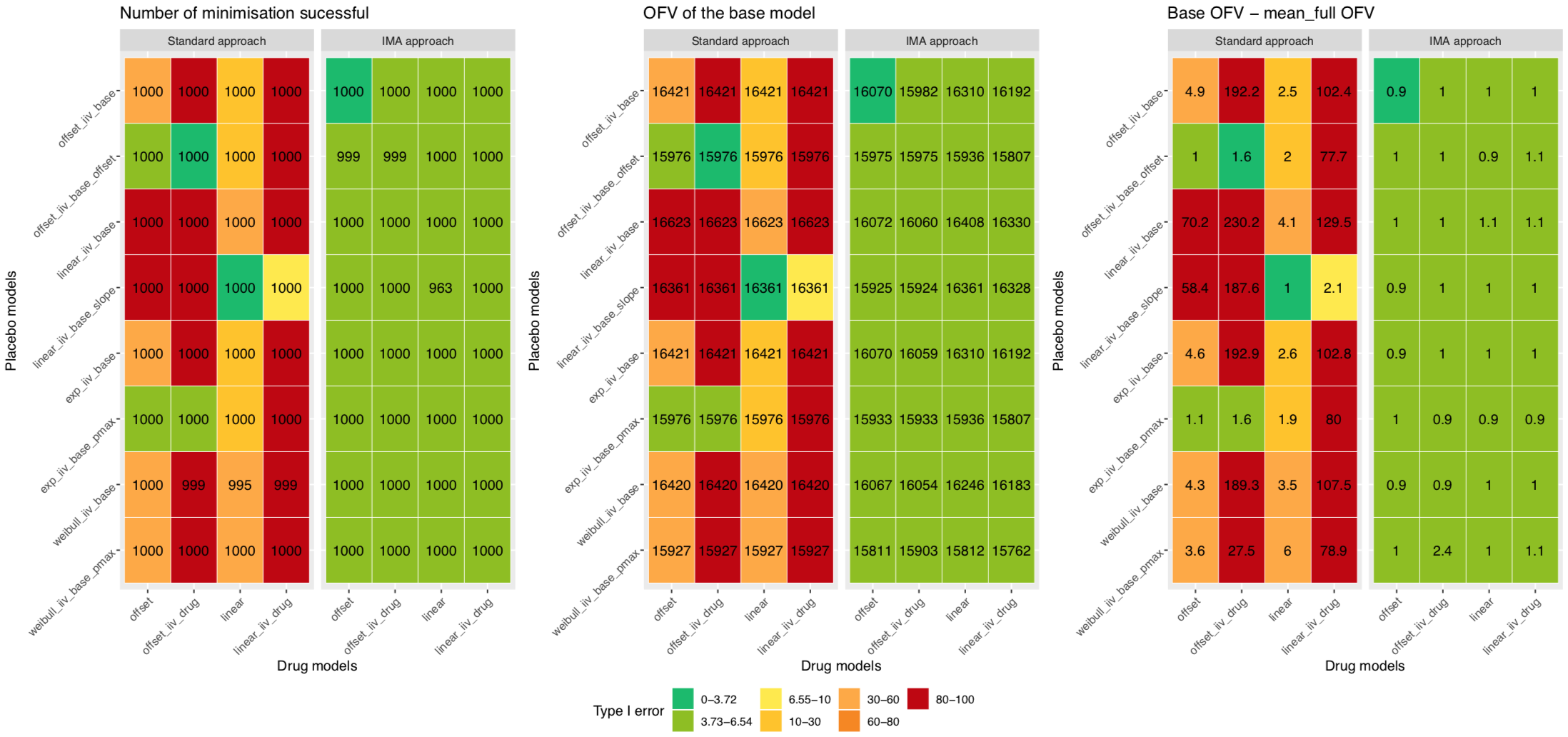


Figure 3: Successful minimization, OFV of the base model, and dOFV between the base OFV and the average full OFV for the seizure count data
