# Supplementary material for: Assessing Treatment Effects with Pharmacometric Models: A New Method that Addresses Problems with Standard Assessments
Source: AAPS J. 2021 May 3;23(3):63. doi: 10.1208/s12248-021-00596-8 (PMC8093168; doi:10.1208/s12248-021-00596-8)
Supplement: Supplementary file 4 — (DOCX 922 kb) [file 12248_2021_596_MOESM4_ESM.docx]

**Supplementary material 4: Results for smaller sample size.**

[Figure 1: Type I error rate with the ADAS-cog data for n=400, n=200, and n=100 individuals 2](#_Toc51009246)

[Figure 2: Type I error rate with the Likert-pain score data for n=60 and n=120 individuals 3](#_Toc51009247)

[Figure 3: Type I error rate with the seizure count data for n=50 and n=250 individuals 3](#_Toc51009248)

Figure 1: Type I error rate with the ADAS-cog data for n=400, n=200, and n=100 individuals

Figure 2: Type I error rate with the Likert-pain score data for n=60 and n=120 individuals

Figure 3: Type I error rate with the seizure count data for n=50 and n=250 individuals
