# Supplementary material for: Assessing Treatment Effects with Pharmacometric Models: A New Method that Addresses Problems with Standard Assessments
Source: AAPS J. 2021 May 3;23(3):63. doi: 10.1208/s12248-021-00596-8 (PMC8093168; doi:10.1208/s12248-021-00596-8)
Supplement: Supplementary file 5 — (DOCX 73 kb) [file 12248_2021_596_MOESM5_ESM.docx]

**Supplementary material 5: Performances of the published models using the standard approach.**

Table 1: Type I error rate of the published models

|  |  | Type I error rate (n=1000) |
| --- | --- | --- |
| ADAS-cog data | + offset drug model | 5.0 % |
|  | + offset drug model with IIV | 18.5 % |
|  | + linear drug model (disease modifying) | 40.3 % |
|  | + linear drug model with IIV (disease modifying) | 98.8 % |
| Seizure count | + offset drug model | 100 % |
|  | + offset drug model with IIV | 100 % |
|  | + linear drug model | 80.2 % |
|  | + linear drug model with IIV | 99.2 % |

Due to run time issues, the published Likert pain score model was run only once with each drug model, hence the dOFV is displayed.

|  |  | OFV (dOFV) |
| --- | --- | --- |
| Likert pain score | No drug model | 48902.15 (Reference) |
|  | + offset drug model | 48902.11 (-0.04) |
|  | + offset drug model with IIV | 48894.13 (-8.02) |
|  | + linear drug model | 48902.11 (-0.04) |
|  | + linear drug model with IIV | 48889.18 (-12.97) |
